# 1039. Clinical and Economic Impact of COVID-19 and Serologic Protection among Farm Workers: Results from the Guatemala Agricultural Workers and Respiratory Illness Impact (AGRI) Study

**DOI:** 10.1093/ofid/ofac492.880

**Published:** 2022-12-15

**Authors:** Daniel Olson, Diva M Calvimontes, Molly Lamb, Kelsey Lesteberg, Edgar Barrios, Neudy Rojop, Anna Chard, Chelsea Iwamoto, Lindsey Duca, Jose Carlos Monzon, Kareen Arias, Melissa Gomez, Claudia Paiz, Eduardo Azziz-Baumgartner, Emily Zielinski Gutierrez, Hani Mansour, Edwards Kathryn, Lee S Newman, David Beckham, Mario Santiago, Edwin J Asturias

**Affiliations:** CU School of Medicine, Denver, Colorado; Fundacion Para La Salud Integr, Los Encuentros, Retalhuleu, Guatemala; Colorado School of Public Health, Aurora, Colorado; CU School of Medicine, Denver, Colorado; Fundacion Para La Salud Integral de los Guatemaltecos, Los Encuentros, Retalhuleu, Guatemala; Fundacion Para La Salud Integral de los Guatemaltecos, Los Encuentros, Retalhuleu, Guatemala; Center for Disease Control and Prevention, Atlanta, Georgia; Center for Disease Control and Prevention, Atlanta, Georgia; Center for Disease Control and Prevention, Atlanta, Georgia; Center for Disease Control and Prevention, Atlanta, Georgia; Fundacion Para La Salud Integral de los guatemaltecos, Los Encuentros, Retalhuleu, Guatemala; Fundacion Para La Salud Integr, Los Encuentros, Retalhuleu, Guatemala; Fundacion Para La Salud Integral de los Guatemaltecos, Los Encuentros, Retalhuleu, Guatemala; Center for Disease Control and Prevention, Atlanta, Georgia; Center for Disease Control and Prevention, Atlanta, Georgia; University of Colorado - Denver, Denver, Colorado; Vanderbilt University, Nashville, Tennessee; Colorado School of Public Health, Aurora, Colorado; CU School of Medicine, Denver, Colorado; CU School of Medicine, Denver, Colorado; CU School of Medicine, Denver, Colorado

## Abstract

**Background:**

In the Guatemala AGricultural workers and Respiratory Impact (AGRI) study, we evaluated the clinical and socioeconomic burdens of respiratory disease in a cohort of Guatemalan banana farm workers.

**Methods:**

All eligible workers were offered enrollment from June 15–December 30, 2020, and annually, then followed for influenza-like illnesses (ILI) through: 1) self-reporting to study nurses, 2) sentinel surveillance at health posts, and 3) absenteeism. Workers with ILI submitted nasopharyngeal swabs for influenza, RSV, and SARS-CoV-2 testing, then completed surveys at days 0, 7, and 28. Enrollment and acute-illness serum samples were tested for anti-SARS-CoV-2 nucleocapsid IgG (anti-N, Roche Elecsys^®^), and neutralizing antibodies (NAb) were tested in a subset using a lentivirus-based pseudovirion assay.

**Results:**

Through October 10, 2021, 1,833 workers were enrolled. The majority were male (84%), young (mean 31 years), and healthy (< 13% had comorbidity). Through October 10, 2021, 1,833 workers developed 169 ILIs (12.0/100 person-years) and 43 (25.4%) of these ILIs were laboratory-confirmed SARS-CoV-2 (3.1/100 person-years). Workers with SARS-CoV-2-positive ILI reported more anosmia (p< 0.01), dysgeusia (p< 0.01), difficulty concentrating (p=0.01), and irritability (p=0.01), and greater clinical and well-being severity scores (Flu-iiQ) than test-negative ILIs (Fig 1); they also had greater absenteeism (p< 0.01) and lost income (median US$127.1, p< 0.01). Among 1334 workers enrolled in 2020, 616 (46.2%) had anti-N IgG suggestive of prior SARS-CoV-2 infection. COVID-19 incidence density for IgG-seropositive workers was 0.4/100 Person – Years (P – Y), lower than those who were seronegative (2.3/100 P – Y) (Fig 2). At enrollment, anti-N IgG titers in serum correlated with neutralizing antibody titers (R^2^=0.26, p< 0.0001). Notably, in < 6 months from enrollment, most workers with follow-up NAb testing (65/77, 84%) exhibited a 95% decrease in neutralizing antibody titers.

**Conclusion:**

Guatemalan farm workers suffered a significant burden of COVID-19, including more severe clinical and economic outcomes than other respiratory illnesses. Ongoing vaccination programs and longitudinal serology will provide additional insight into long-term immunity.

**Disclosures:**

**Daniel Olson, MD**, Pfizer: Grant/Research Support|Roche: Grant/Research Support **Diva M Calvimontes, MD**, Pfizer: Grant/Research Support **Molly Lamb, PhD**, Pfizer: Grant/Research Support **Edwards Kathryn, MD**, Bionet: Advisor/Consultant|IBM: Advisor/Consultant|Merck: Data Monitoring Committee|Moderna: Data Monitoring Committee|Pfizer: Data Monitoring Committee|Roche: Data Monitoring Committee|Sanofi: Data Monitoring Committee|Seqirus: Data Monitoring Committee|X-4 Pharma: Data Monitoring Committee **Edwin J. Asturias, MD**, Curevac: DSMB Member|Fundacion para la Salud Integral de los Guatemaltecos: Board Member|Inovio: DSMB Member|Merck: Honoraria|Pfizer: Grant/Research Support.

